# Timing of anti-VEGF therapy and postoperative macular edema after cataract surgery in eyes with retinal vein occlusion: a retrospective cohort study

**DOI:** 10.1186/s12886-026-05040-z

**Published:** 2026-06-30

**Authors:** Kevin Dahlan, Anthony Oganov, Nicholas Fazio, Khurram M. Chaudhary

**Affiliations:** 1 Department of Ophthalmology, Stony Brook University Hospital, 101 Nicolls Rd, Stony Brook, NY 11794 USA; 2 Department of Ophthalmology, New York Eye and Ear Infirmary of Mount Sinai, New York, NY 10003 USA

**Keywords:** Anti-VEGF therapy, Branch retinal vein occlusion (BRVO), Cataract surgery, Central retinal vein occlusion (CRVO), Cystoid macular edema, Postoperative macular edema, Retinal vein occlusion (RVO)

## Abstract

**Background:**

Patients with retinal vein occlusion (RVO) undergoing cataract extraction are at increased risk of postoperative macular edema (pME), however, optimal perioperative management strategies remain unclear. This study evaluated the incidence of pME in patients with prior RVO undergoing cataract extraction and assessed the effect of anti-VEGF timing on pME outcomes.

**Methods:**

A retrospective cohort study at a single institution was conducted by chart review (2013–2023) using ICD-10 codes for RVO and CPT codes for subsequent cataract extraction in the same eye. Exclusion criteria included diabetic macular edema, lack of perioperative optical coherence tomography, and complex cataract extraction. A total of 53 eyes from 51 patients met study criteria. Data collected included demographics, medical history, ocular history, pre- and postoperative central subfield thickness, average cube thickness, timing of anti-VEGF relative to cataract extraction, fluorescein angiography, and postoperative topical medications. pME was defined as a > 30% increase in CST from baseline. Univariate and multivariate logistic regression were performed to identify independent risk factors for pME.

**Results:**

The cohort had a mean age of 74.3 ± 9.9 years; 56.6% of eyes had branch RVO and 43.4% had central RVO. The overall incidence of pME was 26.1%, with mean time to development of 48.1 ± 25.1 days. A total of 38 eyes (71.7%) had previously received anti-VEGF therapy. Eyes receiving anti-VEGF within 35 days prior to cataract extraction had a significantly lower incidence of pME (12.5%, *n* = 24) compared with those treated more than 35 days before surgery (57.1%, *n* = 14; *p* = 0.033). Among eyes with ischemic RVO on fluorescein angiography, none receiving anti-VEGF within 35 days developed pME, whereas 80.0% of those treated outside this interval developed pME (*p* = 0.002). On multivariate logistic regression, pretreatment with anti-VEGF remained the only significant independent factor (adjusted OR 0.041, 95% CI 0.004–0.466, *p* = 0.010), after adjustment for ischemic status and diabetes.

**Conclusions:**

Patients with RVO have a higher risk of developing postoperative macular edema after cataract extraction. Anti-VEGF pretreatment within 35 days before cataract extraction was associated with lower incidence of pME, particularly in cases of ischemic RVO. These findings suggest that perioperative anti-VEGF timing may influence postoperative outcomes, warranting further prospective investigation.

## Background

Retinal vein occlusion (RVO) is a retinal condition characterized by thrombus formation with partial or total blockage of the retinal veins, leading to dilatation of the retinal veins, subretinal hemorrhages, and retinal ischemia [1]. RVO can be further classified depending on the location of the occlusion as central (CRVO) or in a branch (BRVO). Common risk factors for its development include advanced age and systemic conditions such as hypertension, arteriosclerosis, diabetes, dyslipidemia, and vascular strokes [2].

RVO negatively affects visual acuity (VA), most commonly through macular edema and retinal ischemia. Cystoid macular edema caused by RVO can lead to permanent visual acuity decline and may be treated with anti-vascular endothelial growth factor (anti-VEGF) or corticosteroid injections, which have been shown to improve visual acuity [3, 4]. Ischemic RVO identified on fluorescein angiography (FA) has less favorable outcomes in terms of visual acuity (VA) than non-ischemic RVO [5]. Patients with RVO and concurrent cataract have been shown to benefit from cataract extraction (CE) to significantly improve visual outcomes, although not to the same degree as before RVO [6]. However, CE poses challenges as it significantly increases the risk of postoperative macular edema (pME) in patients with preexisting RVO (2.6%-27.4%) compared to the general population (0.8%-2.4%), leading to potentially permanent decreased post-operative vision gain and prolonged postoperative management [7–9]. Although this risk is greater in patients with RVO, there are currently no standardized perioperative treatments to prevent or reduce pME in patients with RVO.

Compared to CME from RVO, diabetic macular edema (DME) is similar in pathogenesis due to a combination of ischemic and inflammatory mediators, which lead to vascular permeability [10]. In multiple retrospective and placebo-controlled studies, patients with DME who underwent CE benefited from perioperative anti-VEGF injections prior to CE, ultimately reducing pME and improving VA outcomes postoperatively [11–13]. Additionally, prolonged perioperative use of topical non-steroidal anti-inflammatory (NSAID) medications such as ketorolac and nepafenac have shown to improve VA outcomes and pME in patients with diabetic retinopathy. No such improvement was noted after prolonged topical corticosteroid treatment [14, 15].

Previous studies have shown that there was no significant anatomical difference after CE in patients who had active CME from RVO that were concurrently managed with anti-VEGF injections before CE [16]. However, unlike for diabetic retinopathy, a research gap exists as there are currently no guidelines for the necessity, frequency, or temporality of injections in patients with a history of RVO undergoing CE. Similarly, the effectiveness of prolonged topical treatment in the prevention of pME in patients with RVO has not been established.

In this single institution retrospective cohort study, we evaluated the incidence of postoperative macular edema in patients with RVO undergoing CE in the same eye and examined whether the timing of preoperative anti-VEGF therapy influences postoperative outcomes. Specifically, we assessed the relationship between preoperative disease activity and the development of postoperative macular edema, and whether anti-VEGF treatment use and timing prior to surgery may reduce the risk of this complication.

## Methods

**Fig. 1:**
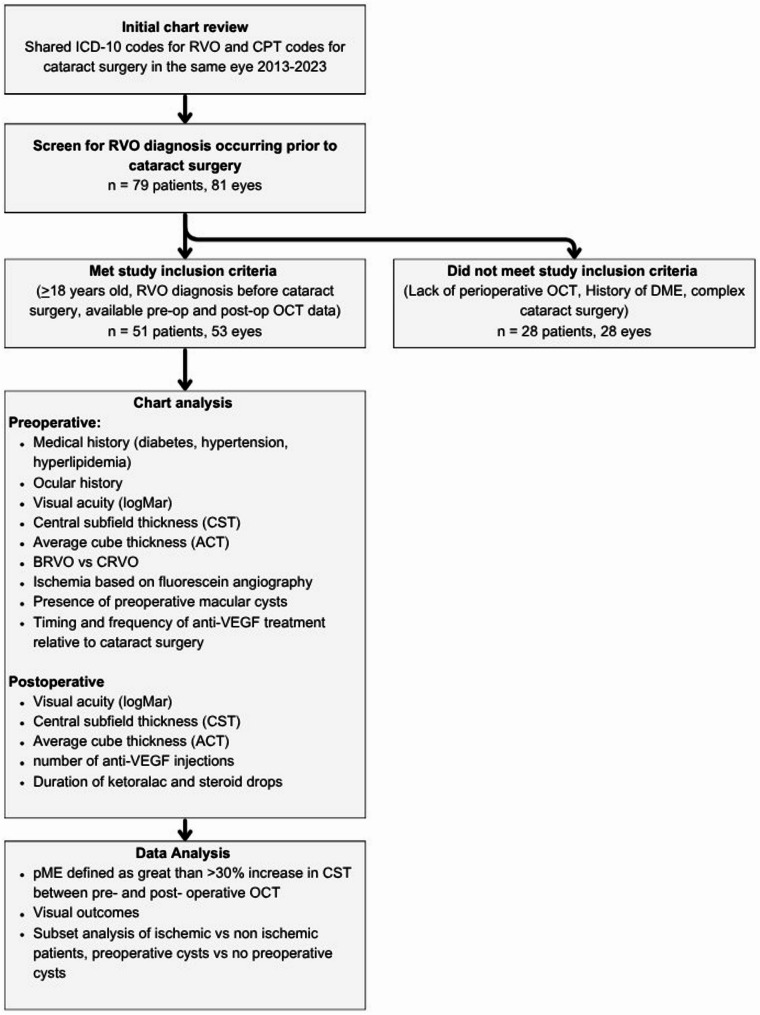
Study workflow

A retrospective chart review was conducted for 81 eyes of 79 patients who shared ICD-10 and ICD-9 codes for RVO (including BRVO and CRVO) and CPT codes for cataract surgery in the same eye between years 2013 and 2023 (Fig. 1). Institutional Review Board (IRB) approval was obtained for the chart review. Informed consent was not sought for the study because of its retrospective nature and the de-identification of patient information. The exclusion criteria included a history of DME, lack of perioperative OCTs, and complicated cataract surgery. In total, 53 eyes of 51 patients were included.

The chart review was based on shared ICD-10 codes for RVO and CPT codes for cataract surgery in the same eye. After screening for RVO diagnosis prior to CE, patients who met the study inclusion criteria underwent subsequent chart analysis, and data analysis was performed based on outcomes.

### Data collection

Data collected and analyzed included patient demographics including age, sex, medical conditions (hypertension, diabetes, hyperlipidemia), ocular history, preoperative and postoperative visual acuity (LogMAR), central subfield thickness (CST), average cube thickness (ACT) on optical coherence tomography (OCT), and timing and frequency of anti-VEGF treatment relative to cataract surgery.

VA gain was reported as the VA difference from before CE to the visit with the thickest CST recorded on OCT (**Δ**VA). pME was defined as a greater than 30% increase in CST between pre- and postoperative OCT as previously described in literature [17]. Patients were stated to have prolonged topical therapy if prednisolone acetate 1% or ketorolac tromethamine 0.5% use was extended beyond the standard 1-month postoperative use.

### Definitions

“Pretreatment” was defined as receiving anti-VEGF injection less than or equal to 35 days prior to CE. The 35-day cutoff was selected to approximate one week beyond the typical four week dosing interval for intravitreal anti-VEGF therapy, allowing evaluation of injections administered within a clinically relevant perioperative treatment window. This interval is pharmacokinetically conservative: reported aqueous humor half-lives are approximately 9–10 days for bevacizumab, 7–9 days for ranibizumab, and 9–11 days for aflibercept in human non-vitrectomized eyes [18–20] (such that all agents would be expected to fall below therapeutic intraocular concentrations well within 35 days).

### Fluorescein angiography grading

Subset analysis among eyes with RVO was performed using fluorescein angiography (FA) interpreted by a single fellowship trained retina specialist as ischemic vs. non-ischemic based on greater or less than 10-disc areas of capillary non-perfusion on wide-field or 7-field fundus FA, consistent with published diagnostic criteria [21, 22].

### Preoperative cyst grading

Another subset of analysis of eyes with RVO was performed for the presence of preoperative macular cysts. Patients with available preoperative OCT images of the operative eye prior to CE were reviewed for the presence of intraretinal cystoid changes. Two independent graders, blinded to postoperative outcomes, classified eyes as having cysts present or absent based on macular OCTs findings.

### Statistical analysis

We performed comparisons of CST, ACT, and LogMAR visual acuity between various patient groups and report the results below. Fisher exact test was used for all categorial binary predictors. Independent-samples t-test for continuous predictors. Linear regression analysis was performed to evaluate the association between proportional change in central subfield thickness (CST) and the time interval to peak CST following cataract extraction. Univariate logistic regression was performed for each predictor. Multivariate logistic regression included covariates of anti-VEGF pretreatment ≤ 35 days, ischemic status on FA, and type 2 diabetes. Diabetes was selected as a covariate given the pathophysiological parallels between DME and RVO associated macular edema, testing whether diabetic eyes were a possible driving effect of edema outcomes. Preoperative cystoid changes could not be included in the multivariate model due to nonconvergence due to small subgroups. Spearman rank correlations were calculated for postoperative BCVA (at peak CST) versus peak CST as well as ΔVA versus peak CST. Statistical significance was defined as *p* < 0.05. Graphical representation of results was made in GraphPad Prism 10. Analyses were conducted in Python 3.12.0.

## Results

### Patient demographics

A total of 81 eyes from 79 patients with a history of RVO later underwent cataract surgery in the same eye. Out of this original cohort, 53 eyes of 51 patients had OCT data available both before and after CE and did not have DME or complicated cataract surgery. Of these 51 patients, the average age at surgery was 74.3 ± 9.9 years, 30 (58.8%) were male and 21 (41.2%) were female (Table 1). The medical history of this group included 35 (68.6%) patients with hypertension, 16 (31.4%) with type 2 diabetes, and 27 (52.9%) with hyperlipidemia. Of the 53 eyes, 30 (56.6%) were classified as having BRVO and 23 (43.4%) as having CRVO. There was no significant association for any demographic or medical comorbidity for eyes that developed pME versus those that did not (all *p* > 0.5).

**Table 1:** Demographic and baseline clinical characteristics

Characteristic	Value
**Patient demographics (** ***n*** ** = 51 patients)**	
Age at surgery, mean ± SD (years)	74.3 ± 9.9
Sex, male, n (%)	30 (58.8%)
**Medical history (** ***n*** = 51 patients*)	
Hypertension, n (%)	35 (68.6%)
Type 2 diabetes, n (%)	16 (31.4%)
Hyperlipidemia, n (%)	27 (52.9%)
**RVO characteristics (** ***n*** ** = 53 eyes)**	
BRVO, n (%)	30 (56.6%)
CRVO, n (%)	23 (43.4%)
FA performed, n (%)	45 (84.9%)
Ischemic on FA, n (% of FA eyes)	17 (37.8%)
**Pre-operative OCT (** ***n*** ** = 53 eyes)**	
Baseline CST, mean ± SD (µm)	276.2 ± 89.4
Preoperative cystoid changes, n (%)	19 (35.8%)
**Anti-VEGF history**	
Prior anti-VEGF, n (%)	38 (71.7%)
Bevacizumab, n (% of injected)	27 (71.1%)
Aflibercept, n (% of injected)	10 (26.3%)
Ranibizumab, n (% of injected)	1 (2.6%)
Treatment naive, n (%)	15 (28.3%)
Pretreated ≤ 35 days, n (% of injected)	24 (63.2%)

*49 out of 51 patients with complete comorbidity data

### Incidence and timing of pME

The incidence of pME in the study population (*n* = 53 eyes) was 26.1%. The average time to pME development was 48.1 ± 25.1 days (range, 7–86 days; *n* = 14). There was no association between the proportional change in CST and the time course to the thickest CST (R²=0.0). There was no association with a proportional change in ACT between any of the groups (*p* > 0.05).

### History and timing of anti-VEGF injections

**Fig. 2:**
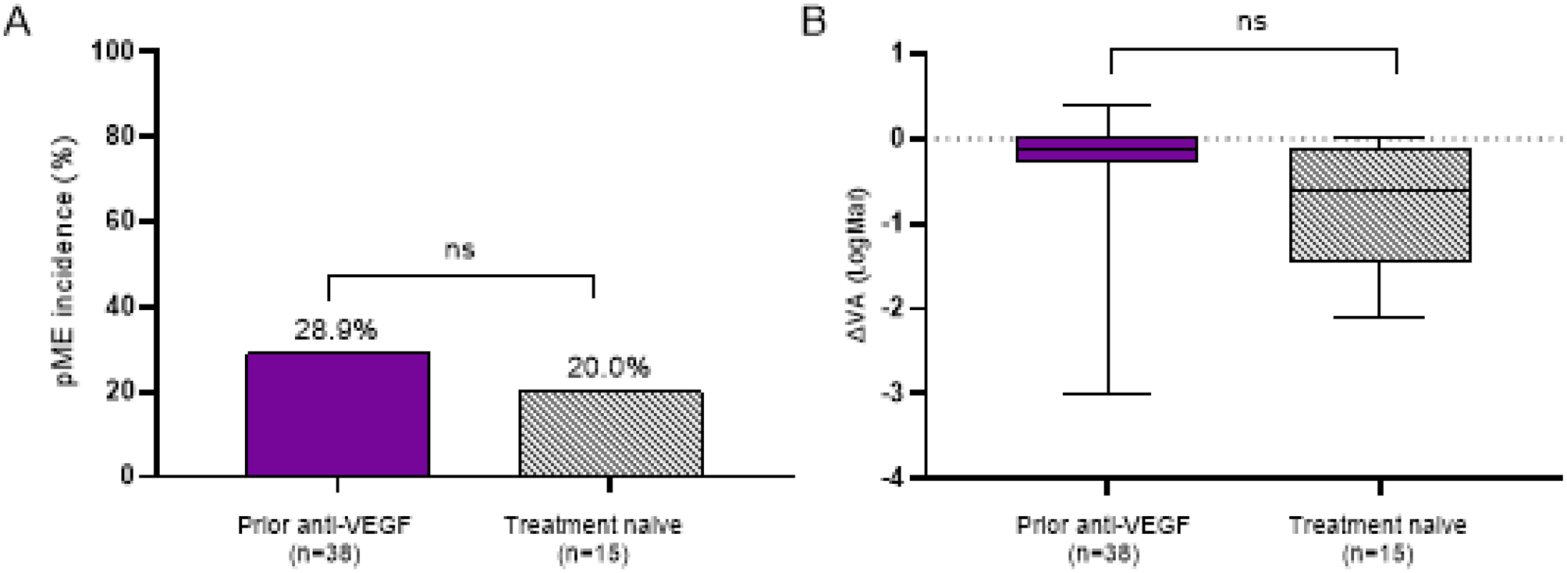
Incidence of postoperative macular edema (pME) and change in Visual acuity in patients with and without prior anti-VEGF injection history. **A**. Incidence (%) of pME following cataract surgery in eyes with a history of anti-VEGF injections (*n* = 38, purple) versus treatment naive eyes (*n* = 15, diagonal shade). No statistically significant difference was observed between groups (ns, not significant). **B**. Change in visual acuity, ΔVA (LogMAR at thickest OCT- LogMAR preoperative), in eyes with prior anti-VEGF (purple) and without prior anti-VEGF injection (diagonal shade) history. No statistically significant differences were observed between groups (ns, not significant)

**Fig. 3:**
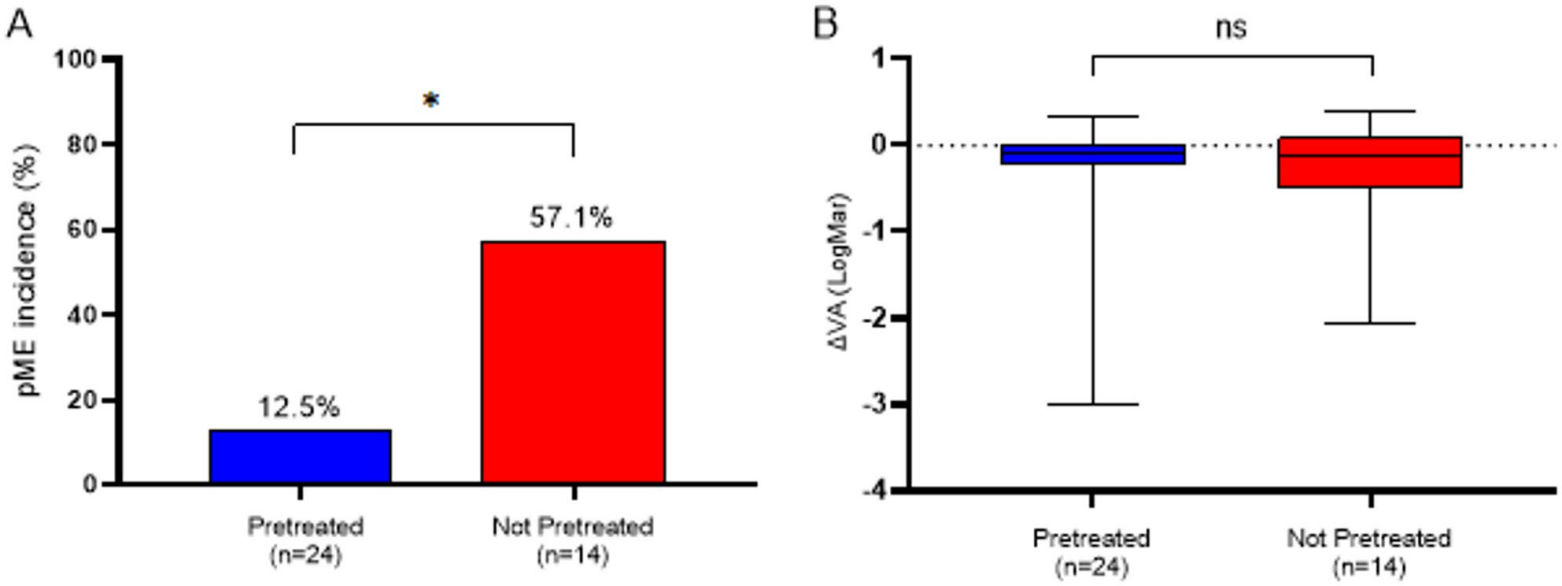
Impact of timing of anti-VEGF injection on incidence of postoperative macular edema (pME). **(A)** Incidence of pME following cataract surgery between eyes pretreated with anti-VEGF within 35 days of surgery (“Pretreated”, *n* = 24, blue) and those not pretreated within that time frame (“Not Pretreated”, *n* = 14, red). Eyes receiving recent anti-VEGF pretreatment (≤ 35 days) had a significantly lower incidence of pME than those injected > 35 days prior to surgery (*p* < 0.05). **(B)** Change in visual acuity, ΔVA (LogMAR at thickest OCT- LogMAR preoperative), in eyes with pretreatment (blue) and without pretreatment with anti-VEGF (red) within 35 days of surgery. No statistically significant differences were observed between groups. (ns, not significant)

Among the 53 eyes, 38 (71.7%) had a history of receiving anti-VEGF prior to CE, and 15 (28.3%) were treatment naive. The incidence of pME was not significantly different between eyes that had previously received anti-VEGF and those that were treatment-naive (28.9% vs. 20.0%; *p* = 0.732) (Fig. 2A). Within the group of eyes that previously received anti-VEGF, those pretreated before CE (*n* = 24) developed pME at a significantly lower incidence of 12.5% compared to those not pretreated before CE at 57.1% (*p* = 0.033) (Fig. 3A).

### Univariate predictors of pME

Among the factors in the univariate analysis for developing pME, anti-VEGF pretreatment ≤ 35 days was the only variable significantly associated (OR 0.11, 95% CI 0.02–0.53, *p* = 0.033) **(**Table 2**).** Ischemic status from FA, preoperative cysts, RVO subtype, and all other comorbidities were non-significant (*p* > 0.05). Continuous variables of age, baseline CST, and pre-operative VA were also all non-significant (*p* > 0.05).

**Table 2:** Univariate analysis of predictors of postoperative Macular Edema

Predictor	Predictor present pME, *n* (%)	Predictor absent pME, *n* (%)	OR (95% CI)	*p*-value
***Binary predictors***				
**Anti-VEGF pretreatment ≤ 35 days** ^a^	***n*** ** = 24: 3 (12.5%)**	***n*** ** = 14: 8 (57.1%)**	**0.11 (0.02–0.53)**	**0.033**
Ischemic RVO on FA^b^	*n* = 17: 5 (29.4%)	*n* = 28: 6 (21.4%)	1.53 (0.38–6.07)	0.722
Preoperative cystoid changes	*n* = 19: 5 (26.3%)	*n* = 34: 9 (26.5%)	0.99 (0.28–3.55)	1.000
BRVO (vs. CRVO)	*n* = 30: 8 (26.7%)	*n* = 23: 6 (26.1%)	1.03 (0.30–3.54)	1.000
Type 2 diabetes^c^	*n* = 16: 3 (18.8%)	*n* = 33: 10 (30.3%)	0.53 (0.12–2.28)	0.502
Hypertension^c^	*n* = 35: 7 (20.0%)	*n* = 14: 6 (42.9%)	0.33 (0.09–1.28)	0.152
Hyperlipidemia^c^	*n* = 27: 7 (25.9%)	*n* = 22: 6 (27.3%)	0.83 (0.23–2.99)	1.000
***Continuous predictors***,*** all 53 eyes (mean ± SD)***				
	**pME Yes (** ***n*** ** = 14)**	**pME No (** ***n*** ** = 39)**	—	**p-value**
Age (years)	73.3 ± 12.4	74.6 ± 8.9	—	0.673
Baseline CST (µm)	248.9 ± 49.1	286.0 ± 98.6	—	0.186
Pre-operative VA (LogMAR)	1.12 ± 0.88	0.86 ± 0.76	—	0.300

Boldface = statistically significant (*p* < 0.05). OR = odds ratio; CI = confidence interval; CST = central subfield thickness; VA = visual acuity; ^a^ Among eyes with prior anti-VEGF history and calculable injection-to-surgery interval (*n* = 38), ^b^ Among 45 eyes with fluorescein angiography data. ^c^ Among 49 eyes with complete comorbidity data

### RVO subtype: ischemic vs. non-ischemic

**Fig. 4:**
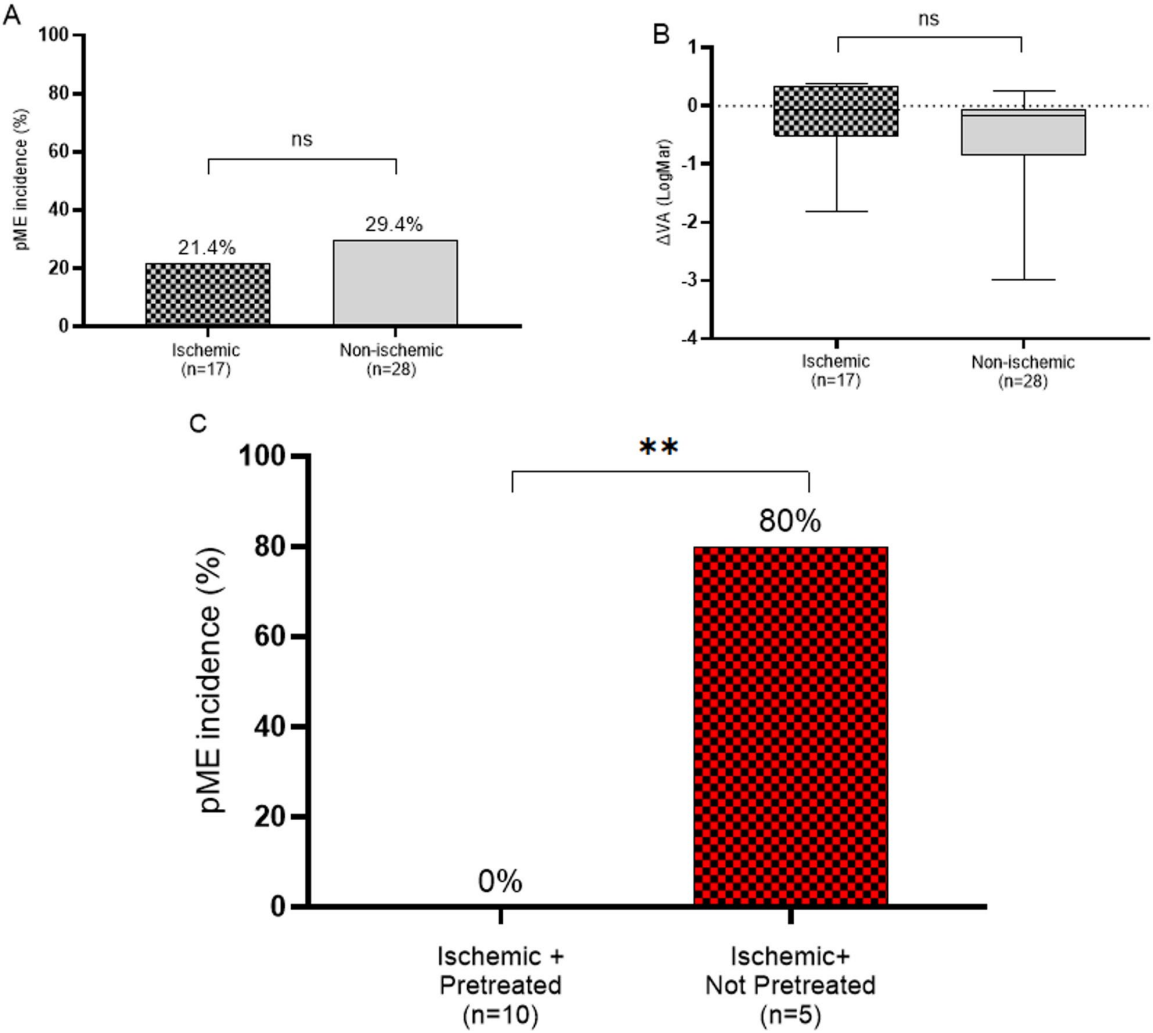
Influence of ischemia and pretreatment on postoperative macular edema (pME) and visual outcomes. (**A**) Incidence of pME (%) following cataract surgery was similar between ischemic (*n* = 17, gray, patterned) and non-ischemic eyes (*n* = 28, gray, non-patterned). (ns, not significant). (**B**) Change in visual acuity, ΔVA, (LogMAR at thickest OCT- LogMAR preoperative), after surgery did not differ significantly between ischemic, -0.40, (*n* = 17, gray, patterned) and non-ischemic eyes, -0.62, (*n* = 28, gray, non-patterned). (**C**) Among eyes with ischemic RVO, those pretreated with anti-VEGF within 35 days of surgery (*n* = 10. blue patterned) had a significantly lower incidence of pME (0.0%) compared to non-pretreated ischemic eyes (80.0%) (*n* = 5, red patterned) (*p* < 0.01)

FA data were available for 45 of the 53 eyes. Seventeen eyes (37.8%) were identified as ischemic, whereas 28 (62.2%) were non-ischemic. No eyes with ischemia on FA that received pretreatment with anti-VEGF developed pME (0.0%), whereas those that received injections outside the pretreatment time had a significantly higher rate of pME at 80.0% (*p* = 0.002) (Fig. 4). We found no significant difference in pME between eyes with ischemia on FA and those without evidence of ischemia on FA (29.4% vs. 21.4%; *p* = 0.92).

### Presence or absence of preoperative cysts

**Fig. 5:**
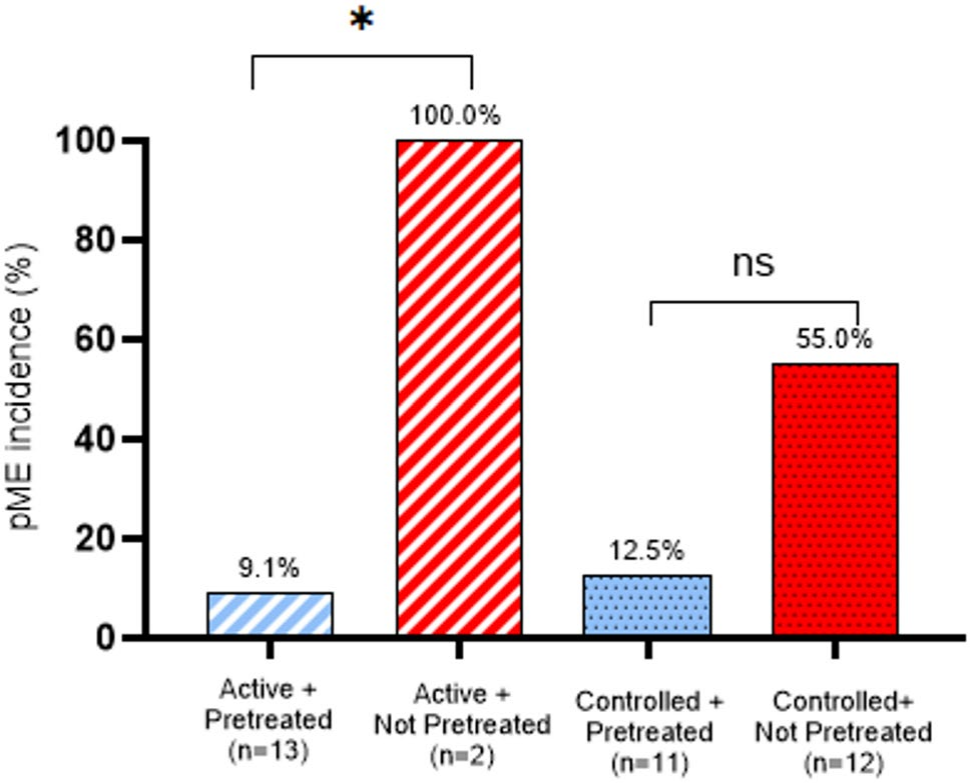
Effect of anti-VEGF pretreatment on postoperative macular edema (pME) stratified by disease activity prior to surgery. Among eyes with active disease (presence of cysts on OCT) and history of anti-VEGF treatment at any time before cataract surgery, those pretreated with anti-VEGF within 35 days (*n* = 13) had lower incidence of pME (9.1%) than non-pretreated eyes (100%) (*n* = 2) (*p* < 0.05). Among eyes with controlled disease (*n* = 23) (no presence of cysts on OCT), no significant difference in pME incidence was observed between the pretreated (12.5%) (*n* = 11) and non-pretreated groups (55%) (*n* = 12) (ns, not significant)

64% of the eyes had controlled disease preoperatively (lack of cysts on OCT), whereas 36% had active disease preoperatively (presence of cysts on OCT). Eyes with active disease and pre-treatment before CE had a pME incidence of 9.1% compared to 100% of those with active disease and no pretreatment (*p* = 0.039) (Table 3) (Fig. 5). Eyes with controlled disease and pre-treatment had a pME incidence of 12.5% compared to 55% of those with controlled disease and no pretreatment (*p* = 0.147).

**Table 3:** Postoperative macular edema incidence by clinical subgroup

Subgroup	*n*	pME, *n* (%)	*p*-value
All eyes	53	14 (26.1%)	—
***Anti-VEGF history***			
Prior anti-VEGF (any)	38	11 (28.9%)	0.732^a^
**Pretreated ≤ 35 days**	**24**	**3 (12.5%)**	**0.033** ^b^
Not pretreated (> 35 days)	14	8 (57.1%)	—
Treatment-naive	15	3 (20.0%)	—
***Ischemia status (FA-graded eyes***, ***n = 45)***			
Ischemic	17	5 (29.4%)	0.920^c^
Non-ischemic	28	6 (21.4%)	—
**Ischemic**,** pretreated ≤ 35 days**	**10**	**0 (0.0%)**	**0.002** ^d^
Ischemic, not pretreated	5	4 (80.0%)	—
***Disease activity (preoperative OCT)***			
Active disease, pretreated	13	1 (9.1%)	0.039^e^
Active disease, not pretreated	2	2 (100.0%)	—
Controlled disease, pretreated	11	1 (12.5%)	0.147^f^
Controlled disease, not pretreated	12	6 (55.0%)	—
***RVO subtype***			
BRVO	30	8 (26.7%)	1.000^g^
CRVO	23	6 (26.1%)	—

“Not pretreated” = prior anti-VEGF > 35 days before CE. Superscripts: ^a^ Injected vs. naive; ^b^ Pretreated vs. not pretreated; ^c^ Ischemic vs. non-ischemic; ^d^ Ischemic pretreated vs. not pretreated; ^e^ Active disease pretreated vs. not pretreated; ^f^ Controlled disease pretreated vs. not pretreated; ^g^ BRVO vs. CRVO. Boldface = statistically significant

### Multivariate predictors of pME

Multivariate logistic regression including pretreatment ≤ 35 days, ischemic status, and diabetes as covariates (*n* = 31, pME events = 8) (Table 4). Anti-VEGF pretreatment ≤ 35 days was the only statistically significant independent predictor of pME (adjusted OR 0.041, 95% CI 0.004–0.466, *p* = 0.010), after adjustment for ischemic status and diabetes. Ischemic status (adjusted OR 2.581, *p* = 0.389) and diabetes (adjusted OR 0.668, *p* = 0.709) were not independently associated with pME risk in this sample.

**Table 4:** Multivariate logistic regression: independent predictors of postoperative macular edema

Predictor	Adjusted OR	95% CI	*p*-value
**Anti-VEGF pretreatment ≤ 35 days**	**0.041**	**0.004–0.466**	**0.010**
Ischemic RVO on FA	2.581	0.299–22.320	0.389
Type 2 diabetes	0.668	0.081–5.544	0.709

Among injected eyes with calculable dates and complete covariate data (*n* = 31, pME events = 8). OR = odds ratio; CI = confidence interval

### Postoperative injections 6 months after CE

Eyes with active disease and pre-treatment before CE had an average of 3.27 anti-VEGF injections within 6 months post-surgery compared to 4.00 among those with active disease and no pretreatment (*p* = 0.779). Eyes with controlled disease and pre-treatment had an average of 3.38 anti-VEGF injections within 6 months post-surgery compared to 2.1 among those with controlled disease and no pretreatment (*p* = 0.250).

### Topical steroid use

Prolonged postoperative topical medication use was documented in 15 of 46 eyes with available data (32.6%), with 31 eyes received standard topical therapy (1 month). Prolonged topical therapy was not associated with a significant reduction in pME incidence compared to standard therapy (20.0% vs. 25.8%; *p* > 0.05). When stratified by pretreatment status, prolonged topical therapy among pretreated eyes showed 0.0% pME (*n* = 9) vs. 16.7% (*n* = 12) with standard therapy, although was non-significant (*p* > 0.05). Among non-pretreated eyes, prolonged topical therapy conferred no additional benefit over standard therapy (60.0% vs. 50.0%) in reducing pME incidence in this sample.

### Visual acuity changes

The average **Δ**VA (LogMAR VA on thickest OCT- LogMAR VA preoperative) in the study population was − 0.425 LogMAR units (*p* = 0.00025), indicating a 4-line increase. ΔVA was not affected in eyes with pME (-0.53) compared to eyes without pME (-0.44) (*p* = 0.72). This result persisted regardless of the injection history (*p* > 0.05) (Fig. 2B). ΔVA between eyes with ischemia on FA (-0.40) was not significant compared to those without evidence of ischemia on FA (-0.62) (*p* = 0.33) (Fig. 4B). There was no significant difference in ΔVA between any of the other subgroups (*p* > 0.05).

**Fig. 6:**
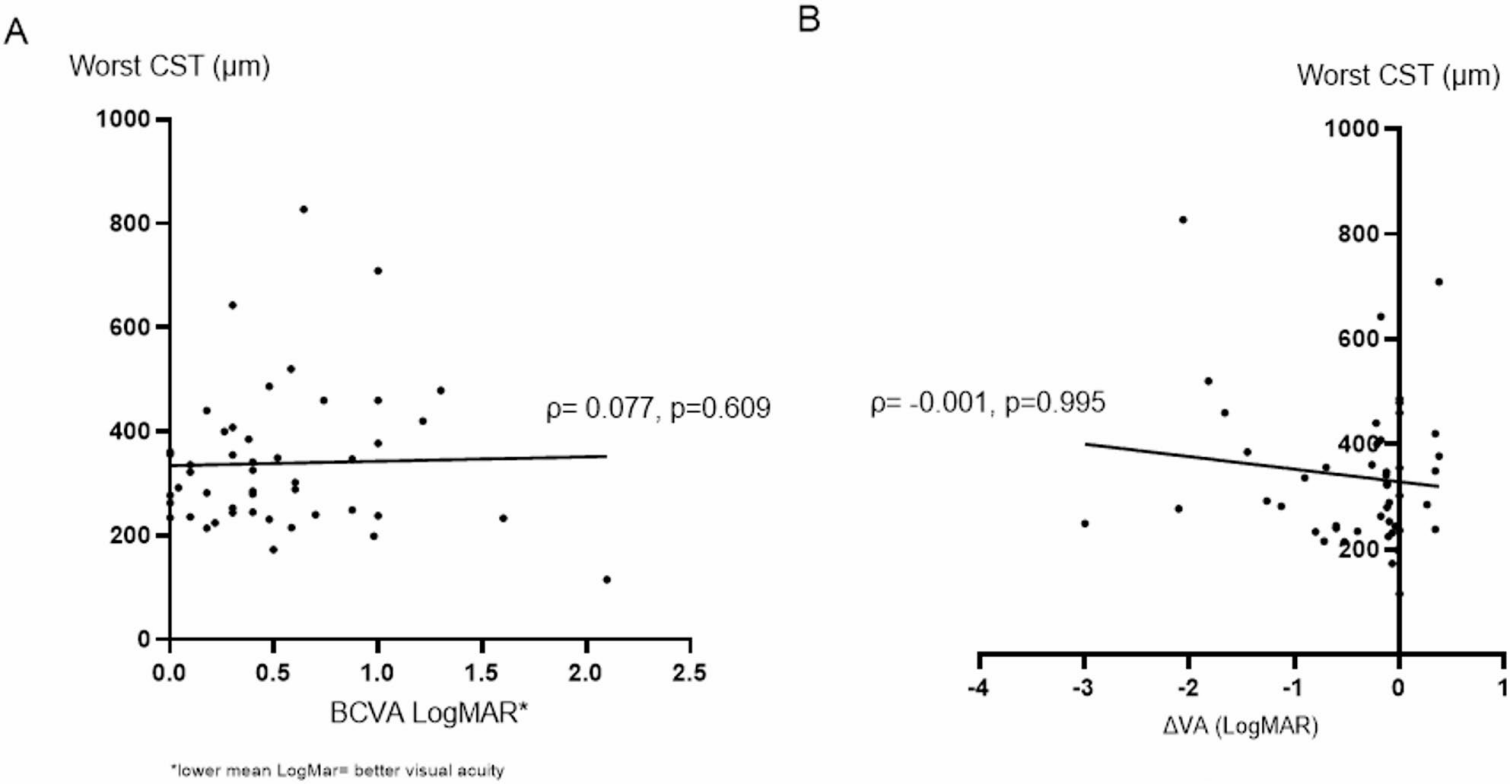
Spearman correlations: peak CST vs. visual acuity outcomes. (**A**) Peak postoperative CST vs. BCVA at worst post-op OCT (ρ = 0.077, *p* = 0.609). Best-fit regression lines shown for reference. No significant relationship between CST elevation and concurrent BCVA. (**B**) Peak postoperative CST vs. ΔVA (ρ=−0.001, *p* = 0.995). Best-fit regression lines shown for reference. No significant relationship between CST elevation and change in visual acuity outcomes

Spearman rank correlation between peak postoperative CST and BCVA at the time of thickest postoperative CST on OCT was ρ = 0.077 (*p* = 0.609), indicating no significant association between the degree of CST elevation and visual acuity at the time of maximal central macular thickening (Fig. 6). Spearman rank correlation between peak postoperative CST and change in visual acuity (postoperative BCVA - preoperative VA) labeled as ΔVA was ρ = −0.001 (*p* = 0.995), showing no significant relationship between the magnitude of postoperative CST elevation and overall visual acuity change following CE.

**Fig. 7:**
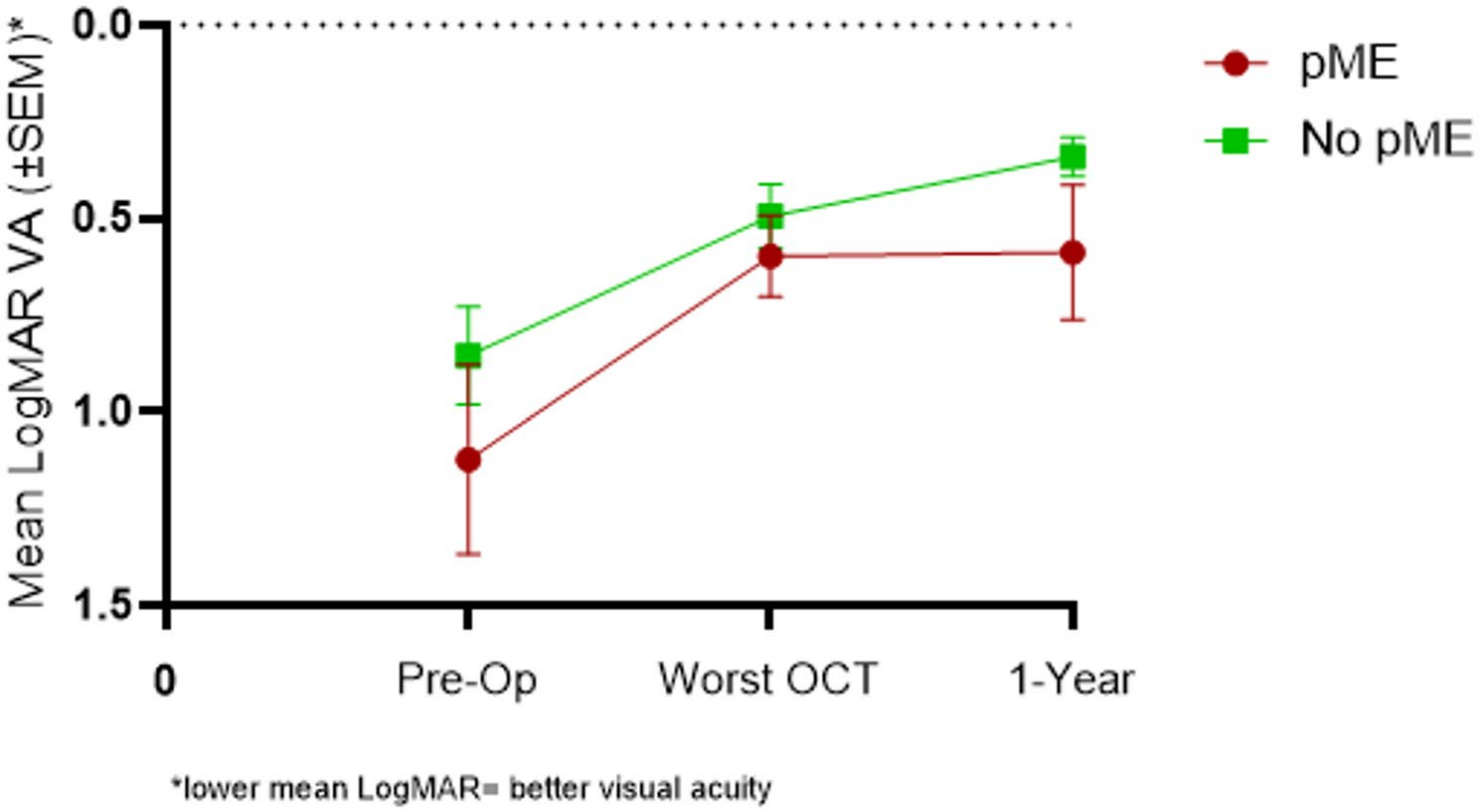
Visual acuity trajectory by pME status. Mean LogMAR VA (± SEM) at pre-operative, worst post-operative OCT, and 1-year timepoints for pME eyes vs. non-pME eyes. 1-year outcomes did not differ significantly between groups (*p* = 0.264). *Lower LogMAR = better visual acuity

Those that developed pME vs. No pME, demonstrated meaningful improvement in logMAR VA from the preoperative baseline to the 1-year follow-up timepoint after CE (Fig. 7). Eyes that developed pME had a mean preoperative LogMAR VA of 1.12 ± 0.88 (SD). No statistically significant difference in VA was observed between pME and non-pME eyes at any timepoint, including at 1-year follow-up (*p* = 0.264). Both groups demonstrated sustained visual improvement from baseline to 1 year, with no evidence of long-term visual deterioration.

## Discussion

### Overview

In this single institution retrospective cohort study, anti-VEGF therapy administered within 35 days prior to cataract extraction was associated with a significantly lower incidence of postoperative macular edema in eyes with retinal vein occlusion. As well, this study demonstrated a relatively higher incidence of pME after CE in eyes with RVO than documented in the general population [7]. This adds to previous studies which have demonstrated pME risk after CE in diabetic patients, as well others showing the inflammatory postoperative period in eyes with RVO also increases the risk of pME [9, 10]. In the multivariate analysis, we controlled for baseline type 2 diabetes to isolate that factor which could contribute to pME, ultimately showing anti-VEGF pretreatment was the only significant risk factor of those analyzed. In our study population, most eyes that received preoperative FA were noted to be non-ischemic, which corresponds to the natural history studies of RVO [22]. The incidence of pME was high in all eyes with RVO, regardless of the level of ischemia on preoperative FA and the previous requirement for anti-VEGF. This high incidence suggests that pre-treatment may be beneficial for all patients, regardless of prior disease activity, to prevent pME.

### Effect of perioperative anti-VEGF timing on postoperative macular edema

In eyes with a history of anti-VEGF treatment, pretreatment with anti-VEGF within 35 days of CE was associated with a significantly lower incidence of pME. This may suggest that preoperative anti-VEGF therapy ameliorates the vascular permeability that leads to pME in eyes with RVO who undergo CE. This finding supports the results of a small study that demonstrated no anatomic difference between preoperative and postoperative CST in eyes with active disease treated regularly with anti-VEGF before CE [16]. This also correlates with the findings from multiple prospective studies in patients with diabetic retinopathy, which found anatomic and VA benefits for perioperative treatment with anti-VEGF prior to CE [11–13]. This suggests that the similar pathophysiology of pME in eyes with RVO and DME appears to respond similarly to peri-operative anti-VEGF treatment [10].

### Influence of ischemic status and fluorescein angiography findings

In our subgroup of eyes with ischemic FAs prior to CE, although the sample size was small, the effect of pretreatment within 35 days of CE was even more pronounced. This may indicate that eyes with severe ischemia may benefit more from perioperative anti-VEGF therapy, suggesting that preoperative FA can be beneficial in identifying RVO eyes that would benefit most from anti-VEGF pretreatment.

### Role of prior anti-VEGF exposure and treatment-naïve eyes

Eyes that had never received anti-VEGF also tended to have a high incidence of pME, which was not statistically different from those who had received anti-VEGF at any time in the past. The absence of a statistically significant difference in any prior anti-VEGF vs. treatment naive suggests that any long term prior anti-VEGF exposure does not grant durable protection against post operative macular edema, and vulnerability of eyes with RVO persistent with or without long term treatment history. Given the retrospective nature of this study, the role of anti-VEGF pretreatment prior to CE in treatment naive patients was not specifically explored in this retrospective study and requires further retrospective investigation among patients receiving their first injection prior to CE, as well as prospective investigation.

### Preoperative disease activity and importance of OCT-based risk stratification

In our comparison of disease activity prior to CE, pretreatment resulted in less edema after CE in all eyes, but was only statistically significant in eyes with active disease. This supports the heightened preoperative monitoring of patients with RVO with OCT prior to CE to identify cystoid changes for risk stratification. Regardless of preoperative disease activity, eyes analyzed required multiple injections on average after CE; therefore, close retinal monitoring after CE is paramount.

### Impact of prolonged topical anti-inflammatory therapy

Extended topical medication treatment with either prednisolone or ketorolac in the postoperative period was not associated with a decrease in pME development in our small study population, however this null result warrants careful interpretation. The role of prolonged topical treatment for pME in eyes with RVO has not been previously investigated. Our findings contrast with recent studies in patients with diabetes, which found that prolonged topical treatment with NSAIDs for 90 days postoperatively with either ketorolac or nepafenac significantly reduced the incidence of pME (2.3%) compared with controls (17.3%) [14, 15]. Although extended use of these medications did not prevent pME in RVO in our small sample, other explanations are also possible. The subgroup of patients who received prolonged topical medications in our study was small and thus may have been underpowered to detect a significant difference. Second generation NSAIDs including bromfenac and nepafenac, which have superior ocular bioavailability were not used. Additionally, the treatment decisions and treatment duration were not randomized and may have been influenced by prolonged clinical postoperative inflammation. Further prospective studies are needed to clarify the role of prolonged use of topical anti-inflammatory medication use in preventing pME in RVO.

### Visual acuity outcomes

In contrast to studies on CME development in postoperative patients, there was no observed difference in **Δ**VA in our study population for those who developed pME [11–13]. One significant confounder that was not controlled for, owing to our retrospective design, was the cataract severity, which may have influenced **Δ**VA independent of pME development. Future prospective studies may reduce this confounding factor by using a potential acuity meter to compare expected versus observed visual acuity gain and isolate the effect of pME, independent of cataract severity. Spearman correlation analysis between peak CST and BCVA at the visit was negligible, showing CST elevation did not significantly alter functional outcomes in our small cohort. Confounding factors may include the varying severity of pME, prompt use of anti-VEGF therapy once pME was discovered, as well as improvement of media clarity from cataract exchange.

### Study limitations

Our study has some limitations, which stem from its retrospective nature. The eyes were not randomized to receive injections, and there was no standardized injection protocol or timing of administration prior to CE. Instead, eyes were statistically divided based on a cutoff of 35 days, which was chosen to represent 1 week after the standard minimal 4-week injection anti-VEGF injection cycles. Anti-VEGF drug heterogeneity (bevacizumab, aflibercept, ranibizumab) represents an uncontrolled confounding variable, though all three agents have intravitreal half-lives within 35 days. For grading of ischemic status on FA, inter rater reliability data was not available as only one retina specialist reviewed the images. The decision to provide prolonged topical treatment (prednisolone acetate 1%, ketorolac tromethamine 0.5%) was not standardized as dosing frequency for prolonged topical medications was not uniformly captured and may have introduced a bias. Our multivariate model non converged on analysis of more factors than anti-VEGF pretreatment, ischemic status, and diabetes most likely due to low patient counts. The patients requiring preoperative OCT imaging may indeed represent a higher risk or surveillance group, potentially overestimating the incidence of pME. Additionally, providers may have obtained more OCT imaging if vision gain was not as expected after CE, potentially including patients with worse disease control. One-year VA data was available for only 34/53 eyes (64.2%). Future prospective studies with standardized protocols for injection timing, OCT imaging intervals, use of topical anti-inflammatory drops, and preoperative visual potential, are needed to further characterize the efficacy of anti-VEGF pretreatment for all patients with a history of RVO prior to CE and to identify patients at the greatest risk of pME.

## Conclusions

In this single institution retrospective study, patients with RVO demonstrate a high incidence of pME after CE, regardless of disease activity level. Eyes that were pretreated with anti-VEGF before CE had a significantly lower incidence of pME than those that did not. In eyes with ischemic RVO classified by FA, this relationship was particularly evident. Prolonged post-operative topical steroid or NSAID treatment did not show a decrease in pME in this small population. Further prospective studies are required to investigate the utility of anti-VEGF pretreatment for the prevention of pME in patients with ischemic and non-ischemic RVO.

## Data Availability

The datasets generated and analyzed during the current study are not publicly available due to institutional privacy regulations but are available from the corresponding author on reasonable request.

## Abbreviations

ACT: Average cube thickness
anti-VEGF: Anti–vascular endothelial growth factor
BRVO: Branch retinal vein occlusion
CE: Cataract extraction
CME: Cystoid macular edema
CRVO: Central retinal vein occlusion
CST: Central subfield thickness
DME: Diabetic macular edema
FA: Fluorescein angiography
ICD: International Classification of Disease
IRB: Institutional Review Board
logMAR: Logarithm of the minimum angle of resolution
OCT: Optical coherence tomography
pME: Postoperative macular edema
RVO: Retinal vein occlusion
VA: Visual acuity
ΔVA: Change in visual acuity
